# Antibiotic susceptibility and clonal distribution of *Staphylococcus aureus* from pediatric skin and soft tissue infections: 10-year trends in multicenter investigation in China

**DOI:** 10.3389/fcimb.2023.1179509

**Published:** 2023-07-13

**Authors:** Wei Su, Ying Liu, Qing Wang, Lin Yuan, Wei Gao, Kai H. Yao, Yong H. Yang, Lin Ma

**Affiliations:** ^1^ Department of Dermatology, Beijing Children’s Hospital, Capital Medical University, National Center for Children’s Health, Beijing, China; ^2^ Laboratory of Dermatology, Beijing Pediatric Research Institute, Beijing Children’s Hospital, Capital Medical University, National Center for Children’s Health, Beijing, China; ^3^ Department of Dermatology, Children’s Hospital Affiliated to Capital Institute of Pediatrics, Beijing, China; ^4^ Department of Dermatology, Children's Hospital Affiliated to Zhengzhou University, Zhengzhou, China

**Keywords:** SSTIs, *Staphylococcus aureus*, MRSA, antimicrobial sensitivity, molecular epidemiology, China

## Abstract

**Background:**

Skin and Soft Tissue Infections (SSTIs) Surveillance Network of *S. aureus* In Pediatrics in China was established in 2009 to routinely report epidemiological changes. We aimed to monitor the present antibiotic sensitivity and molecular characteristics of *S. aureus* and methicillin-resistant *S. aureus* (MRSA) from SSTIs in children nationwide and track the changes over the past decade.

**Methods:**

Patients diagnosed with SSTIs from the dermatology departments of 22 tertiary pediatric hospitals in seven geographical regions of China were recruited continuously from May 2019 to August 2021. *S. aureus* was isolated, and its sensitivity to 15 antimicrobials was evaluated using the broth microdilution method. The molecular characteristics of the MRSA isolates were determined through multilocus sequence typing (MLST) and staphylococcal cassette chromosome *mec* (SCC*mec*) typing. The presence of the Panton–Valentine leukocidin gene (*pvl*) was determined.

**Results:**

The detection rate of *S. aureus* was 62.57% (1379/2204), among which MRSA accounted for 14.79% (204/1379), significantly higher than the result in previous study in 2009-2011 (2.58%, 44/1075). Compared with previous study, the sensitivity to cephalosporins and fusidic acid decreased to varying degrees, while that to chloramphenicol, ciprofloxacin, clindamycin, erythromycin, gentamicin, penicillin, and tetracycline increased significantly. The sensitivity to mupirocin, trimethoprim/sulfamethoxazole (TRISUL), and rifampicin still maintained at a high level (97.90%, 99.35% and 96.66% respectively). The leading multidrug resistance pattern of MRSA and methicillin-sensitive *S. aureus* (MSSA) were erythromycin-clindamycin-tetracycline (55.84%; 43/77) and erythromycin-clindamycin-chloramphenicol (27.85%, 44/158) respectively. 12 high-level mupirocin-resistant strains were detected, and notable differences in geographical distribution and seasonal variation were observed. The main types of MRSA were ST121 (46.08%, 94/204), followed by ST59 (19.61%, 40/204). SCC*mec* V (65.69%, 134/204) and SCC*mec* IV (31.86%, 65/204) were dominant epidemic types. ST121-V, ST59-IV, and ST22-V were the most prevalent clones nationwide. The detection rate of *pvl* had increased markedly from 9.09% (4/44) in 2009-2011 to 22.55% (46/204) in 2019-2021 (P<0.05).

**Conclusion:**

The antibiotic sensitivity and molecular characteristics of *S. aureus* from pediatric SSTIs has changed significantly over the past decade. To standardize medical care, provide timely and reasonable clinical treatment, and effectively manage infection control, Chinese pediatric SSTIs guidelines are urgently needed.

## Introduction

*Staphylococcus aureus* is the main pathogen that causes skin and soft tissue infections (SSTIs), such as pustules, folliculitis, boils, and abscesses, in pediatric patients (Lowy, 1998) as well as fatal infections, such as necrotizing fasciitis and toxic shock syndrome (Burnham and Kollef, 2018). Methicillin-resistant *S. aureus* (MRSA) has attracted considerable attention owing to its drug resistance and virulence (Lee et al., 2018). Community-associated MRSA (CA-MRSA) mainly causes SSTIs in young and healthy people in the community (Turner et al., 2019). Over the past decade, CA-MRSA has been considered to be the main cause of the increased burden of MRSA diseases. Some CA-MRSA strains have been increasingly involved in nosocomial infections and have even become dominant strains in medical settings (Elston and Barlow, 2009). The resistance rate of CA-MRSA is increasing, not only to β-lactam antibiotics but also to non-β-lactam antibiotics (Guo et al., 2020). The spectrum of CA-MRSA invasive diseases is expanding and is increasingly becoming the focus of global infection (Hassoun et al., 2017). Epidemiological information on MRSA is important for clinical decision-making and public health monitoring. Furthermore, classification of MRSA is an important part of describing epidemiological trends and formulating infection control strategies (Mediavilla et al., 2012).

To our knowledge, few studies on the antibiotic sensitivity of *S. aureus* and molecular characteristics of MRSA from SSTIs in China have been conducted (Xiao et al., 2019; Zhao et al., 2022). Furthermore, results from different regions differ, and national studies related to children are rare. A national SSTIs surveillance network of *S. aureus* in pediatrics, established in 2009 and led by the Department of Dermatology, Beijing Children’s Hospital, is the only available nationwide epidemiological surveillance network with regular investigation in China (Liu et al., 2016). Currently, 22 children’s hospitals have joined. The present study aimed to track the changes in antibiotic sensitivity of *S. aureus* as well as the molecular characteristics and epidemiology of MRSA in children diagnosed with SSTIs in China.

## Materials and methods

### Patient enrollment

This was a multi-center, cross-sectional epidemiological study on children diagnosed with SSTIs in the dermatology departments of 22 tertiary pediatric hospitals. According to their geographical location, the hospitals were divided into seven groups: North, Middle, East, South, Northeast, Northwest, and Southwest China. All patients who met the criteria for SSTIs were recruited continuously from the dermatology departments from May 2019 to August 2021. The inclusion criteria were as follows: 1) no history of major congenital malformations or severe chronic diseases, 2) no history of surgery or hospitalization within the past year, 3) no history of dialysis or deep catheterization, and 4) no history of antibiotic use within the past week. Information, including sex, age, predisposing factors, disease type, specimen collection time, and basic medical history, was collected. Patients who could not provide general information were excluded. The enrolled patients were treated according to routine treatment, and the swab specimens of infection sites from the non-repetitive participants were collected continuously.

### Strain identification

The isolated strains were identified using traditional microbial identification methods, coagulase and catalase tests, and latex slide agglutination test (Oxoid Ltd., Basingstoke, UK). All three tests were positive for *S. aureus* (Weist et al., 2006).

### Identification of MRSA strains

In addition to resistance to cefoxitin and oxacillin, MRSA was further identified through polymerase chain reaction (PCR) amplification of the *nuc* and *mecA* genes according to the method described previously (Sahebnasagh et al., 2014). ATCC 29213 and ATCC35601 were used as a negative and positive control, respectively, for the *mecA* gene.

### Susceptibility profiles

The antibiotic susceptibility profiles of the *S. aureus* isolates were determined using the Sensititre^®^ Antimicrobial Susceptibility Testing System (Thermo Scientific, UK), following the manufacturer’s instructions. The minimum inhibitory concentration (MIC) to 15 antibiotics (cefazolin, ceftriaxone, cefuroxime, chloramphenicol, ciprofloxacin, clindamycin, erythromycin, fusidic acid, gentamicin, mupirocin, penicillin, rifampicin, tetracycline, TRISUL, and vancomycin) were detected using the broth microdilution method (Novy et al., 2014). *S. aureus* ATCC 29213 and ATCC 35601 were used for quality control. The antimicrobial sensitivity breakpoints were interpreted according to the current Clinical and Laboratory Standards Institute (CLSI) breakpoints for *S. aureus* (CLSI, 2019), while sensitivity to cefazolin, ceftriaxone, and cefuroxime was interpreted according to a previous version of CLSI (Clinical and Laboratory Standards Institute/NCCLS, 2005). An E-test ((bioMérieux, Marcy-L’Étoile, France) was further performed on the isolates classified as mupirocin-resistant through broth microdilution.

### Epidemiological typing of MRSA

DNA was extracted for PCR using a bacterial genomic DNA extraction kit (Tiangen Biochemical Technology, China). Multilocus sequence typing (MLST) was performed on MRSA isolates using the method described previously (Enright et al., 2000). Sequences of seven housekeeping genes (*arcC*, *aroE*, *glpF*, *gmk*, *pta*, *tpi*, and *yqiL*) were compared with known alleles from the MLST database (https://pubmlst.org/organisms/staphylococcus-aureus). Allelic profiles and sequence types (STs) were determined using the database.

The isolates were also subjected to staphylococcal cassette chromosome *mec* (SCC*mec*) typing, which is based on multiplex PCR with 10 primers (Omuse et al., 2016). SCC*mec* types I-V were assigned according to the combination of the cassette chromosome recombinase (*ccr*) type and *mec* class. Isolates that could not be assigned to any expected type were defined as non-typable.

### Panton–Valentine leukocidin gene detection

*pvl* was amplified using PCR as described previously (Hesje et al., 2011) with minor modifications. MRSA N315 was used as a negative control, while ATCC 25923 was used as a positive control.

### Statistical analysis

A database including the age, sex and disease patterns of the patients, antimicrobial resistance and molecular characteristics of the corresponding isolate was constructed in Microsoft Excel 2003. GraphPad Prism 9.0 (GraphPad Software Inc., San Diego, CA, United States) was used to create graphs. All susceptibility data were analyzed using WHONET software (version 5.6). JMP^®^ 11 Statistical Discovery Software (S.A.S. Institute Inc., Cary, North Carolina) was used for statistical analysis. Categorical variables were analyzed using the chi-squared (χ2) or Fisher’s exact test. Significance was set at *P* < 0.05.

## Results

### General information

**Figure 1:**
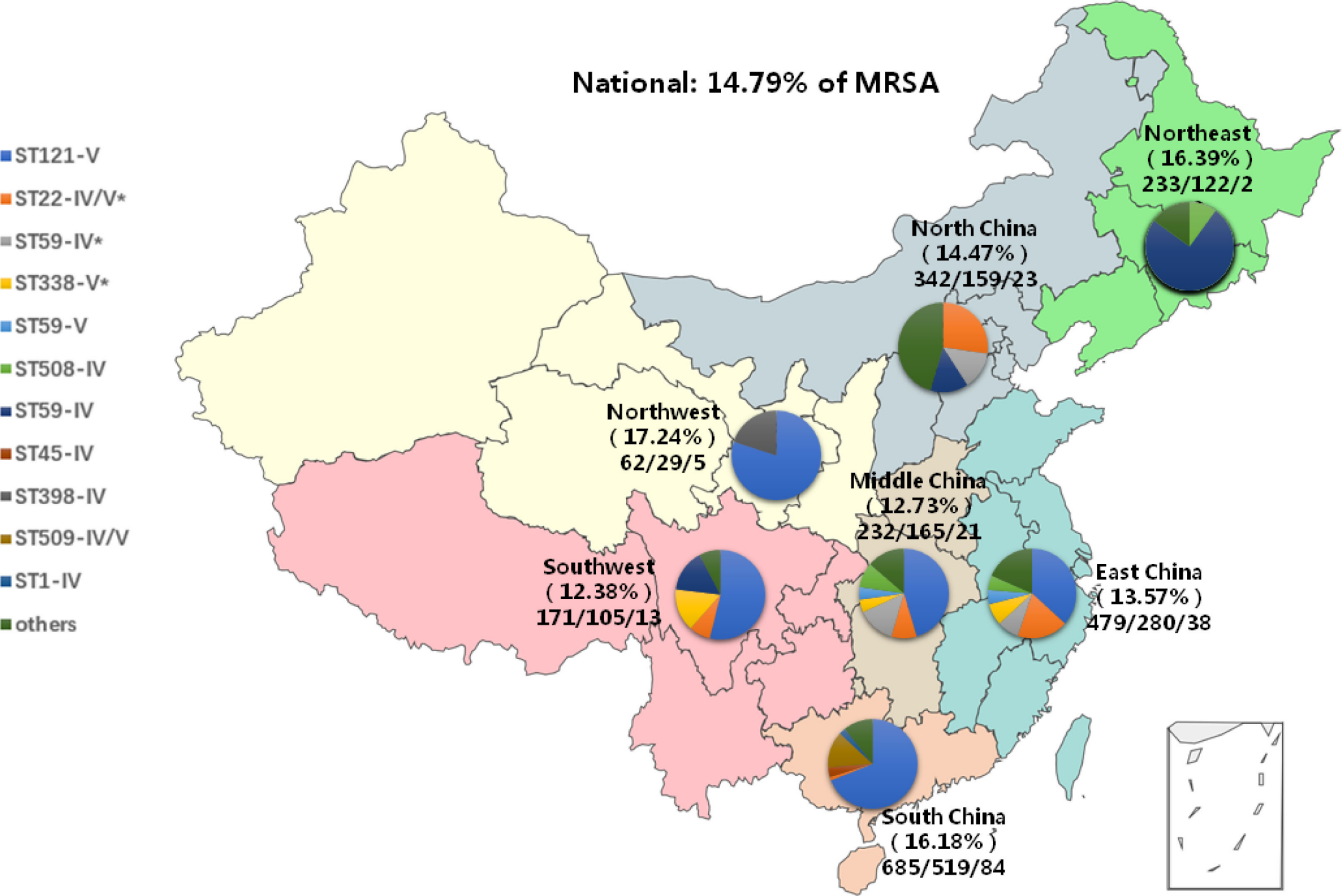
The distribution of specimens, *S. aureus* and MRSA as well as prevalent clones, by region. All seven geographical regions were included as follows: Northeast, green, North China, gray; East China, blue; Northwest, yellow; Middle China, khaiki; Southwest, pink; South China, orange. *pvl-positive clones contained.

The initial data of *S. aureus* and MRSA collection as well as the distribution of them are summarized here. From May 2019 to August 2021, 2204 patients with SSTIs were enrolled in 22 children’s hospitals in 19 provinces of seven geographic regions. The overall positive rate of *S. aureus* was 62.57% (1379/2204). The detection rate of *S. aureus* was the highest at 75.77% (519/685) in South China and was the lowest at 46.49% (159/342) in North China. The proportion of MRSA was 14.79% (204/1379) nationally, with significantly different proportions across China ranging from 12.73% (21/165) in Middle China to 17.24% (5/29) in North West China. Single institutional prevalence ranged from 4.35% (1/23) to 30.77% (8/26) (*P* < 0.05). The distribution of enrolled patients and the number of positive *S. aureus* and MRSA isolates in each geographic region are shown in **Figure 1**.

The clinical features of patients from whom the strains were collected are analyzed as follows. The age of patients ranged from 3 days to 18 years old, with 77.16% (1064/1379) aged 1–6 years. Of the patients, 58.45% (806/1379) were males and 41.55% (573/1379) were females. Detailed clinical features are presented in **Table 1**.

**Table 1:** Clinical features of patients from children with SSTIs in the present study.

Variable	Numbers (%)	Region	Hospital	Patients/SA/MRSA (n)
**General information**		**East China**		
**Total patients**	2204	Anhui province	AHCH	96/74/13
**SA**	1379 (62.57%)	Zhejiang province	HZCH	100/58/8
MSSA	1175 (85.21%)	Shandong province	JNCH	52/23/1
MRSA	204 (14.79%)	Jiangsu province	NJCH	36/21/2
**Sex**		Jiangsu province	XZCH	100/78/6
Male	806 (58.45%)	Zhejiang province	ZJCH	95/26/8
Female	573 (41.55%)	**Middle China**		
**Ages**		Hubei province	HBMCHH	34/18/2
0-28 days	11 (0.80%)	Hunan province	HNCH	98/61/12
1-12 months	167 (12.11%)	Henan province	ZZCH	100/86/7
1-3 years	401 (29.08%)	**North China**		
3-6 years	496 (35.97%)	Beijing	BJCH	140/50/10
6-12 years	278 (20.16%)	Inner Mongolia province	NMGCH	95/47/5
12-18 years	26 (1.89%)	Beijing	CIOPCH	107/62/8
**Types of SSTIs**	**SA/MRSA**	**North East**		
Primary infection	1059/155	Jilin province	CCCH	99/50/15
Impetigo	543/75	Liaoning province	DLCH	97/60/5
Furuncle	274/42	Heilongjiang province	HBCH	37/12/0
Folliculitis	176/28	**North West**		
Abscess	36/7	Shaanxi province	XACH	62/29/5
Paronychia	16/2	**South China**		
SSSS	11/1	Guangdong province	SZCH	332/281/48
Acne	2/0	Guangxi province	LZMCHH	94/60/7
Omphalitis	1/0	Guangdong province	GZCC	161/115/18
Secondary infection	320/49	Hainan province	HNMCHH	98/63/11
Secondary infection of eczema	177/19	**South West**		
Secondary infection of trauma	75/16	Sichuan province	CDCH	90/68/7
Secondary infection of herpes	36/8	Yunnan province	KMCH	81/37/6
Secondary infection of fungi	7/2			
Others	25/4			

MRSA, methicillin-resistant Staphylococcus aureus; MSSA, methicillin-sensitive S. aureus; SA, Staphylococcus aureus.

Dalian Children’s Hospital of Dalian Medical University, DLCH; Children’s Hospital of Changchun, CCCH; Harbin Children’s Hospital, HBCH; Beijing Children’s Hospital, BJCH; Children's Hospital Affiliated to Capital Institute of Pediatrics, CIOPCH; Inner Mongolia maternal and Child Health Hospital, NMGCH; Xuzhou Children’s Hospital, XZCH; Children's Hospital Affiliated to Zhejiang University, ZJCH; Anhui children's Hospital, AHCH; Nanjing Children's Hospital, NJCH; Qilu Hospital Jinan children's Hospital, JNCH; Hangzhou Children's Hospital, HZCH; Xi'an Children's Hospital, XACH; Hubei Maternal and Child Health Hospital, HBMCHH; Hunan Children’s Hospital, HNCH; Zhengzhou Children’s Hospital, ZZCH; Chengdu Children's Hospital, CDCH; Kunming children's Hospital, KMCH; Guangzhou Women and Children’s Medical Center, GZCC; Hainan maternal and Child Health Hospital, HNMCHH; Guangxi Liuzhou Maternal and Child Health Hospital, LZMCHH; Shenzhen Children's Hospital, SZCH.

**Figure 2:**
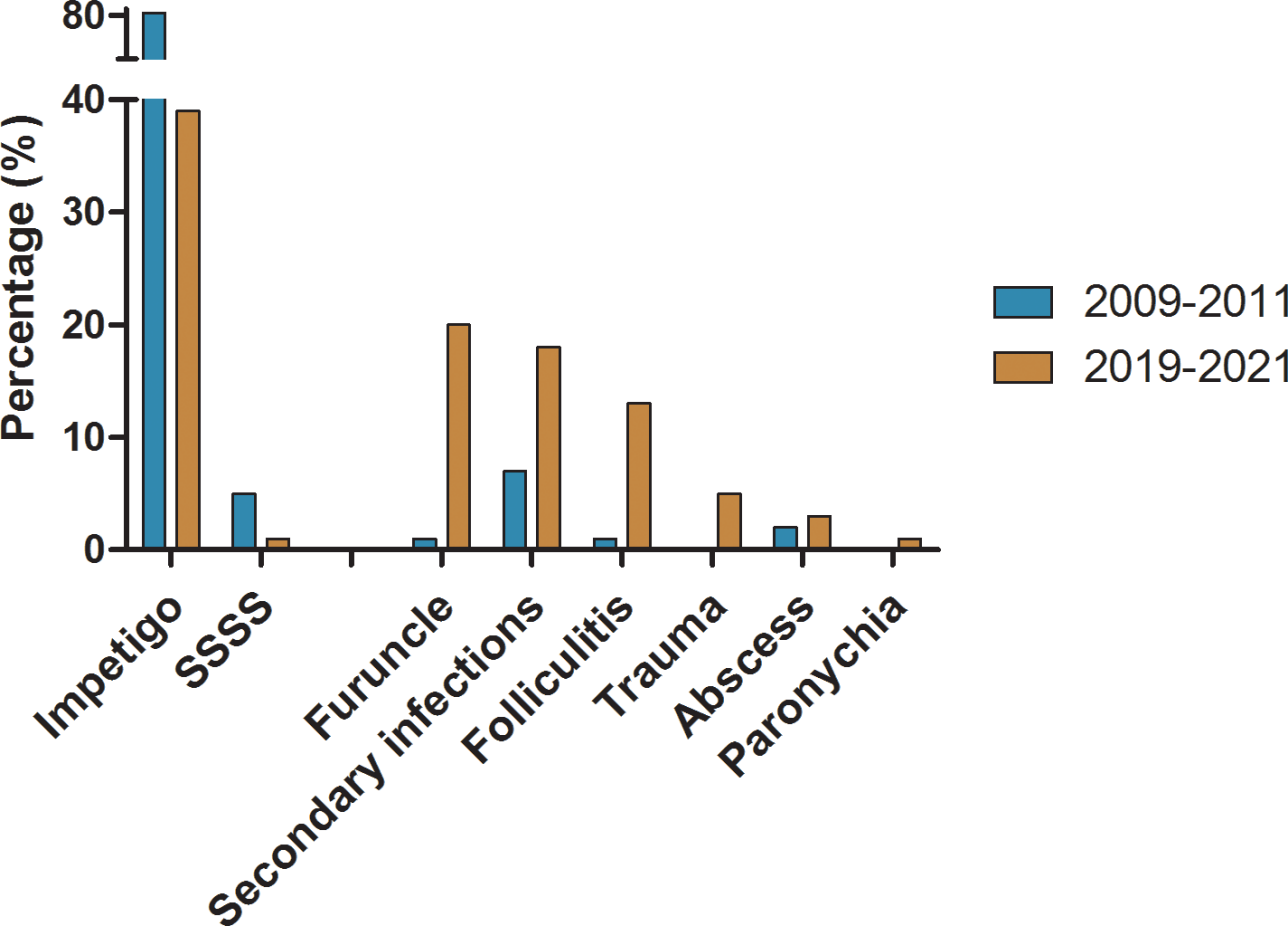
The comparation on distribution of major infection pattern caused by *S. aureus* in 2009-2011 and 2019-2021 respectively.

Great changes on infection patterns had occurred during the past decade. The top three primary infections in this study were impetigo (39.38%; 543/1379), furuncles (19.87%; 274/1379), and folliculitis (12.76%; 176/1379). Compared with our study conducted in 2009-2011 (Liu et al., 2016), the distribution of deep infections such as folliculitis and furuncle in 2019-2021 increased significantly from 1.20% (21/1749) to 12.76% (176/1379) and from 0.57% (10/1749) to 19.87% (274/1379) with *P*<0.001, respectively. The distribution of superficial infections such as impetigo and staphylococcal scalded skin syndrome had decreased from 79.87% (1397/1749) to 39.38% (543/1379) and from 4.69% (82/1749) to 0.80% (11/1379) with *P*<0.001, respectively. The detailed composition of infections caused by *S. aureus* in 2009-2011 and 2019-2021 is shown in **Figure 2**.

### Characteristics of resistance pattern of *S. aureus* and change trend

The results of the antimicrobial susceptibility test on strains collected in this present study are shown in **Table 2**. The resistance rates of MRSA to ciprofloxacin, clindamycin, erythromycin, and tetracycline were significantly higher than those of MSSA (*P*<0.05).

**Table 2:** Results of the antimicrobial sensitivity of the isolates in the present study.

Antimicrobial agent	MRSA (n=204)					MSSA (n=1175)				
	Sensitivity (%)	Resistance (%)	MIC_50_ (μg/mL)	MIC_90_ (μg/mL)	MIC_range_ (μg/mL)	Sensitivity (%)	Resistance (%)	MIC_50_ (μg/mL)	MIC_90_ (μg/mL)	MIC_range_ (μg/mL)
CEFAZO	88.24	11.76	2	>8	1~>8	99.15	0.85	1	1	≤0.25~>8
CEFTRI	49.51	50.49	16	64	6~>256	98.21	1.79	4	4	≤0.25~>16
CEFURO	46.08	10.78	8	32	≤4~>64	98.38	0.34	≤4	≤4	≤4~64
CHLORA	47.55	11.27	16	>32	≤4~>32	55.74	7.32	8	16	≤4~>32
CIPROF	93.14	4.41	≤0.25	0.5	≤0.25~>4	92.00	1.53	≤0.25	0.5	≤0.25~>4
CLINDA	31.86	66.18	>16	>16	≤0.5~>16	47.40	49.96	2	>16	≤0.5~>16
ERYTH	5.39	87.74	>64	>64	0.032>64	14.98	75.91	>64	>64	0.125>64
FUSIDA	97.06	2.94	≤0.125	0.25	≤0.125~>2	95.15	4.85	0.25	0.25	≤0.125~>2
GENTAM	97.55	1.47	≤0.125	0.25	≤0.125~>32	96.60	1.79	≤0.125	0.25	≤0.125~>32
MUPIRO	96.57	2.45	≤0.125	0.25	≤0.125~>512	98.13	1.19	≤0.125	≤0.125	0.06~>512
PENICI	0	100	>8	>8	0.12~>8	6.81	93.19	8	>8	≤0.125~>8
RIFAMP	93.63	0.49	≤0.015	1.5	≤0.015~4	97.19	0.43	≤0.015	0.5	0.002~>32
TETRA	65.20	34.80	≤0.5	32	≤0.5~32	90.04	9.36	≤0.5	2	≤0.5~>32
TRISUL	99.51	0.49	≤0.125	1	≤0.125~8	99.32	0.68	0.5	1	≤0.125~16
VANCOM	100.00	0.00	≤0.5	1	≤0.5~2	100.00	0.00	≤0.5	1	≤0.5~2

CEFAZO, Cefazolin; CEFTRI, cefatriaxone; CEFURO, Cefuroxime; CHLORA, Chloramphenicol; CIPROF, Ciprofloxacin; CLINDA, Clindamycin; ERYTH, Erythromycin; FUSIDA, Fusidic acid; GENTAM, Gentamicin; MUPIRO, Mupirocin; PENICI, Penicillin; RIFAMP, Rifampin; TETRA, Tetracycline; TRISUL, Trimethoprim-sulfamethoxazole; VANCOM, Vancomycin.

*S. aureus* with resistance to three or more classes of antimicrobial agents were defined as multidrug-resistant (MDR). In this study, MDR was observed in 37.75% (77/204) of MRSA strains and 13.45% (158/1175) of MSSA strains. The predominant resistance patterns of MRSA to non-β-lactam antibiotics were erythromycin-clindamycin-tetracycline (55.84%; 43/77), followed by erythromycin-clindamycin-tetracycline-chloramphenicol (18.18%; 14/77). The resistance patterns of MSSA were very different from that of MRSA, the most common profiles of which were erythromycin-clindamycin-chloramphenicol (27.85%, 44/158) and erythromycin- clindamycin-tetracycline (22.15%, 35/158). Among different STs of MRSA strains, the proportion of MDR in ST121 was the highest (49.35%; 38/77), followed by ST59 (22.08%; 17/77) and ST338 (6.49%; 5/77).

A total of 12 high-level mupirocin-resistant (MuH) strains (MIC ≥ 512 μg/mL) were detected, including nine MSSA strains and three MRSA strains. The differences in ST distributions of MuH strains were irregular, while notable in the differences in geographical distribution and the seasonal variation. MuH strains mainly distributed in South China (66.67%, 8/12)、East China (16.67%, 2/12) and Middle China (16.67%, 2/12). Of the hospitals, the isolates were predominantly separated from Shenzhen Children’s Hospital (41.67%, 5/12), which was geographically assigned to South China. The infection patterns were mainly secondary infection, including secondary infection of eczema (5/12), trauma (2/12) and herpes (1/12). The infections caused by MuH isolates mainly occured in autumn (8/12), followed by summer (4/12). The children mainly aged >3y (66.67%, 8/12).

**Figure 3:**
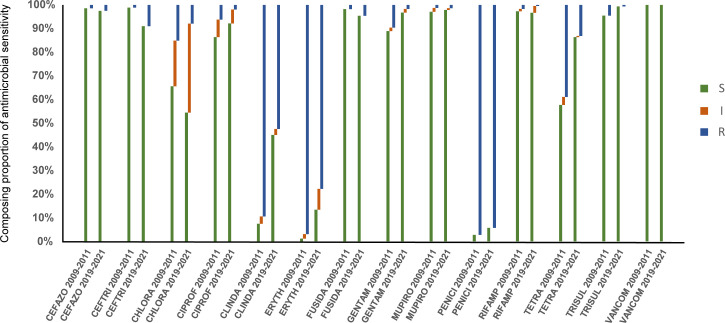
Comparison of antimicrobial susceptible, intermediate and resistant rates of *S. aureus* isolates in 2009-2011 and 2019-2021. S, susceptible; I, intermediate; R, resistant.

The changes of resistance patterns of *S. aureus* collected in this study with strains collected in 2009-2011were analyzed. The current resistance rates of *S. aureus* to cefazolin, ceftriaxone, cefuroxime, and fusidic acid had increased significantly (*P*<0.05), and that to chloramphenicol, ciprofloxacin, clindamycin, erythromycin, gentamicin, rifampicin, tetracycline, and TRISUL decreased significantly (*P*<0.0001), while no significant difference was found in resistance to vancomycin and mupirocin. Besides, the resistance rate to penicillin decreased from 96.8% to 94.13% (*P*=0.0004). The comparison of the antimicrobial sensitivity of *S. aureus* isolates between 2009-2011 and 2019-2021 is shown in **Figure 3**.

### Molecular characteristics of MRSA isolates

Overall, 25 STs were detected, of which ST121 accounted for 46.08% (94/204), followed by ST 59 (19.61%, 40/204) and ST22 (9.80%, 20/204). In SCC*mec* classification, 65.69% (134/204) were type V, and 25.98% (53/204) were type IV. ST121-V, ST59-IV, and ST22-V were the most prevalent clones nationwide. The molecular biological characteristics of MRSA isolates and the dominant MRSA clones by region are summarized in **Table 3**; **Figure 1**.

**Table 3:** Molecular biological characteristics of MRSA isolates in the present study.

CC	MLST	n	*pvl*		SCC*mec*			
			+	-	IV	V	NT	
CC121	ST121	94		94		94	
	ST488	1		1		1	
CC59	ST59	40	14	26	31	6	3
CC22	ST22	20	19	1	5	15	
CC45	ST508	5		5	5		
	ST45	3		3	3		
CC1	ST1	3		3	3		
	ST834	1		1	1		
	ST4855	1		1	1		
CC8	ST72	1		1	1		
	ST630	1		1		1	
CC5	ST6	1		1	1		
CC30	ST30	1	1		1		
NT	ST509	11		11	6	5	
	ST338	7	7			7	
	ST88	2	1	1	1		1
	ST398	1		1		1	
	Others	11	4	7	6	4	1
Total		204	46	158	53	134	17

MLST, multilocus sequence typing; pvl, panton-valentine leukocidin gene; SCCmec, Staphylococcal cassette chromosome mec; NT, non-typable.

+, postive; -, negative.

### Major features of ST121 and ST59 strains

ST121 was the most prevalent type of MRSA strains, which all typed as SCC*mec* V and *pvl* negative. ST121 strains were mainly distributed in Northwest and South China with a positive rate of 80.00% (4/5) and 69.05% (58/84), respectively, while they were not detected in Northeast China. Compared with non-ST121 isolates, ST121 isolates had a significantly higher resistance rate to clindamycin and lower resistance rates to cefazolin, cefuroxime, and ciprofloxacin. The detection rate of ST59 was second to that of ST121. ST59, which was opposite to ST121, was mainly distributed in Northeast and North China, with a positive rate of 80.00% (16/20) and 40.91% (9/22), respectively. Inconsistent with ST121, ST59 strains were mainly typed as SCC*mec* IV (77.50%, 31/40). The positive rate of *pvl* was 35.00% (14/40), significantly higher than that of ST121 (0.00%, 0/94). The resistance rate of ST59 isolates to ciprofloxacin, cefazolin, and cefuroxime was significantly higher than that of ST121 isolates (*P*<0.05), and there was no significant difference in the resistance rate to other antibiotics.

### Clinical and molecular characteristics of *pvl*-positive MRSA strains

**Figure 4:**
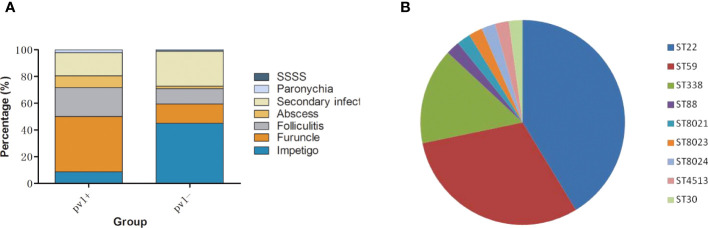
Clinical and molecular characteristics of *pvl*-positive MRSA strains. **(A)**, infection patterns caused by *pvl*-positive and *pvl*-negative MRSA strains. **(B)**, ST distribution of *pvl*-positive MRSA strains.

The detection rate of *pvl* had increased markedly from 9.09% (4/44) in 2009-2011 to 22.55% (46/204) in 2019-2021 (P<0.05). Infection patterns caused by *pvl*-positive and *pvl*-negative MRSA strains in this study are shown in **Figure 4A**. *pvl*-positive MRSA strains mainly caused furuncle (41.30%,19/46) and folliculitis (21.74%,10/46), higher than *pvl*-negative strains with P<0.0001 and P=0.073 respectively, while *pvl*-negative MRSA strains mainly caused impetigo (44.94%,71/158) and secondary infection (25.95%,41/158), higher than *pvl*-positive strains with P<0.0001 and P=0.067 respectively. Among the *pvl*-positive strains, ST22 (41.30%, 19/46), ST59 (30.43%, 14/46) and ST338 (15.22%, 7/46) were the most common types as shown in **Figure 4B**, which were significantly higher than the ratio of *pvl*-negative strains with P<0.05 respectively. In contrast, among *pvl*-negative strains, ST121 (59.49%,94/158) was the most prevalent ST, the ratio of which was significantly higher than that of *pvl*-positive strains (P<0.0001).

## Discussion

This study was the second to conduct national multicenter epidemiological monitoring of *S. aureus* for SSTIs in pediatrics since the surveillance network was established in China in 2009. The epidemiological trends of SSTIs in 22 tertiary children’s hospitals in seven geographical regions of mainland China were investigated. Compared with the study conducted 10 years ago, we presented four major findings: 1) the resistance profiles of *S. aureus* isolates had changed considerably; 2) the prevalence of CA-MRSA and its *pvl*-positive strains, increased significantly; 3) the proportion of deep infections increased significantly; and 4) ST121-V was the dominant clone, with the percentage increased.

In this study, MRSA accounted for 14.79%, significantly higher than in 2009-2011 (2.58%). It was reported that antimicrobial agents should be chosen to target MRSA and MSSA if MRSA accounts for >10% of *S. aureus* among SSTIs (David and Daum, 2017). Either clindamycin or TRISUL was recommended because of the low cost and activity against community-associated MRSA and MSSA strains of each of these drugs (Williams et al., 2011). According to previous studies, one of these antimicrobials should be used in addition to incision and drainage for a skin infection (Miller et al., 2015; Daum et al., 2017). In this study, we identified a low resistance rate of *S. aureus* to TRISUL (0.4%–2%). The long-term use of TRISUL remains a suitable option for treating complex hyperimmunoglobulin E syndrome and chronic granulomatous disease (Hashemi et al., 2017). Clindamycin was reported to be effective at treating infections caused by susceptible CA-MRSA isolates (Miller et al., 2015). However, in this study, though significantly decreased compared with that in 2009-2011, the resistance rate of clindamycin was still higher than 50%, indicating that it is not a good choice for treating SSTIs in children in China.

For empiric or targeted therapy for *S. aureus*, an anti-*staphylococcal* β-lactam drug was the most appropriate choice (David and Daum, 2010). Furthermore, it was reported that cephalosporins and penicillin are most commonly used in China (Li et al., 2016). Therefore, the MICs of the clinical strains to penicillin as well as cephalosporins was detected to track changes in sensitivity. The resistance rates of *S. aureus* to penicillin, though decreased significantly, remained at a high level (94.13%). On the contrary, the sensitivity to cephalosporins was maintained at a high level (90.65%–97.53%). According to this result, cephalosporins might be a suitable alternative to penicillin for empiric therapy.MDR to non-β-lactam antibiotics was detected in both MRSA and MSSA. The presence of MDR strains in outpatients with SSTIs can lead to persistent or recurrent MRSA infections (Lee et al., 2017).

Clinically, most SSTIs can be controlled only with topical antibiotics. Mupirocin is a topical antibiotic that has been extensively used for treating MRSA skin and soft-tissue infections, decreasing certain types of surgical site infections and eliminating nasal colonisation of MRSA among patients and medical staff. In the present study, the sensitivity to mupirocin was still high, consistent with previous results (Liu et al., 2016; Chen et al., 2020). However, high-level mupirocin strains were detected in both MRSA and MSSA strains in this study. Dadashi et al. reported that the incidence of high-level mupirocin resistance in MRSA was the highest in Asia (12.1%), followed by Europe (8.0%) and the USA (5.9%) (Dadashi et al., 2020). In China, it was reported that *mupA* mainly accounted for high level mupirocin resistance (Jin et al., 2018; Guo et al., 2023). The *mupA* gene is typically located on mobile genetic elements and is plasmid mediated, which maybe the reasons for transmission of clones (Liu et al., 2010). In this study, high-level mupirocin resistant strains were mainly isolated from South China, which were generally with higher economical levels than others. Easy access to antibiotics without prescriptions, a high rate of antibiotic misuse, and the frequency of empiric treatment in these regions may lead to the situation. The results suggested that the rational use of mupirocin should be strengthened, and drug resistance should be further monitored.

Fusidic acid was also an important choice for SSTIs. The worldwide resistance rate of *S. aureus*, especially MRSA, to fusidic acid was reported to be 0.3%–64% (Gajdács, 2019). In the present study, the resistance rate of *S. aureus* to fusidic acid increased from 1.8% to 4.57%, which was still low, similar to that identified previously (Gu et al., 2016). The resistance rate in MSSA (4.85%) was higher than that in MRSA (2.94%), consistent with previous study (Zhanel et al., 2021). The increased detection rate of fusidic acid-resistant strains suggests that the drug should be used in moderation.

Based on the above results of antimicrobial sensitivity, we call for the timely introduction of guidelines for the treatment of SSTIs in children in China to develop scientific and effective diagnosis and treatment programs.

MRSA has been the focus of global SSTIs (Mediavilla et al., 2012). Recently, an upward trend in the incidence of *pvl*-positive MRSA was observed in Europe and Japan (Bouchiat et al., 2017; Nakaminami et al., 2020). Concern was raised as *pvl*-positive MRSA strains usually cause deep infections such as furuncles and abscesses (Shallcross et al., 2013). Compared with the study conducted 10 years ago, the detection rate of MRSA in the present study had increased by 5.65-fold (14.79% vs. 2.58%; *P*<0.0001), and the positive rate of *pvl* had increased by 2.3-fold (22.55% vs. 9.8%; *P*<0.05). In addition, the infection spectrum had changed, as deep infections including folliculitis, furuncle, and abscesses increased significantly, while superficial infections decreased. Therefore, according to our surveillance, there was an increasing trend in the prevalence of *pvl*-positive MRSA among SSTI isolates in Chinese children, which was probably connected with the increase in deep infections. Attention should be paid to the surveillance of *pvl-*positive MRSA in the future.

MLST is a universal method for understanding the molecular epidemiology of MRSA (Enright et al., 2000). Previous studies demonstrated that the most prevalent clones of CA-MRSA from SSTIs had unique geographic distribution, as ST8 was mostly reported in the USA (Otter and French, 2010), while ST80 was mainly in Europe, and ST59 was mainly in the Asia–Pacific region (Deurenberg and Stobberingh, 2008). In mainland of China, Taiwan, and Hong Kong, ST59 was reported as the most prevalent ST of MRSA strains from SSTIs (Yu et al., 2015), while ST121 was rarely dominant for clinical infections (Chuang and Huang, 2013; Wang et al., 2019). The epidemiological data hint that most ST121 strains were MSSA (Goering et al., 2008; Rao et al., 2015). However, in the present survey, ST121 (46.08%; 94/204) was the dominant ST in MRSA strains, followed by ST59, which was consistent with the results of our previous study (Liu et al., 2016). This was probably due to the differences in the population. In the present study, the enrolled children were at preschool age (1–6 years). It was reported that among preschool children, ST121 was the most prevalent clone in China (Fan et al., 2009). Besides, they were all outpatients who had no history of hospitalization. Therefore, the study was more representative of infections from the community.

In conclusion, tremendous changes in the antibiotic sensitivity of *S. aureus* from SSTIs in Chinese children had been observed compared with the results obtained 10 years ago. The incidence of MRSA as well as the positive rate of *pvl* had increased significantly, with ST121, ST59, and ST22 being the main epidemic types. With the significant changes, further research tracking sensitivity to antibiotics as well as the molecular epidemiological characteristics of MRSA is needed. Moreover, to standardize medical care, help clinicians make evidence-based treatment decisions, and effectively manage infection control, guidelines for SSTIs in pediatrics in China are urgently needed.

## Data availability statement

The original contributions presented in the study are included in the article/supplementary materials. Further inquiries can be directed to the corresponding author.

## Ethics statement

This project (2019-k-123) was approved by the Research Ethics Committee in Beijing Children’s Hospital, China on May 21, 2019.

## Author contributions

YL, KY, YY and LM designed the study. WS, YL, QW, LY and WG conducted the experiments. WS, YL and QW collected and analyzed the data, interpreted the results, and drafted the manuscript. All authors contributed to the article and approved the submitted version.

## References

[B1] Bouchiat C. Curtis S. Spiliopoulou I. Bes M. Cocuzza C. Codita I.. (2017). MRSA infections among patients in the emergency department: a European multicentre study. J. Antimicrob. Chemother. 72 (2), 372–375. doi: 10.1093/jac/dkw431 27798212

[B2] Burnham J. P. Kollef M. H. (2018). Treatment of severe skin and soft tissue infections: a review. Curr. Opin. Infect. Dis. 31 (2), 113–119. doi: 10.1097/QCO.0000000000000431 29278528 PMC6200137

[B3] Chen W. He C. Yang H. Shu W. Cui Z. Tang R.. (2020). Prevalence and molecular characterization of methicillin-resistant *Staphylococcus aureus* with mupirocin, fusidic acid and/or retapamulin resistance. BMC Microbiol. 20 (1), 183. doi: 10.1186/s12866-020-01862-z 32600253 PMC7325228

[B4] Chuang Y. Y. Huang Y. C. (2013). Molecular epidemiology of community-associated meticillin-resistant *Staphylococcus aureus* in Asia. Lancet Infect. Dis. 13 (8), 698–708. doi: 10.1016/S1473-3099(13)70136-1 23827369

[B5] Clinical and Laboratory Standards Institute/NCCLS (2005). “Performance standards for antimicrobial susceptibility testing; fifteenth informational supplement,” in CLSI/NCCLS document M100-S15 (940 West Valley Road, Suite 1400, Wayne, Pennsylvania 19087-1898 USA: Clinical and Laboratory Standards Institute).

[B6] CLSI (2019). “Performance standards for antimicrobial susceptibility testing,” in CLSI supplement M100, 29th ed, vol. 2019. (Wayne, PA: Clinical and Laboratory Standards Institute).

[B7] Dadashi M. Hajikhani B. Darban-Sarokhalil D. van Belkum A. Goudarzi M. (2020). Mupirocin resistance in *Staphylococcus aureus*: a systematic review and meta-analysis. J. Glob Antimicrob. Resist. 20, 238–247. doi: 10.1016/j.jgar.2019.07.032 31442624

[B8] Daum R. S. Miller L. G. Immergluck L. Fritz S. Creech C. B. Young D.. (2017). A placebo-controlled trial of antibiotics for smaller skin abscesses. N Engl. J. Med. 376 (26), 2545–2555. doi: 10.1056/NEJMoa1607033 28657870 PMC6886470

[B9] David M. Z. Daum R. S. (2010). Community-associated methicillin-resistant *Staphylococcus aureus*: epidemiology and clinical consequences of an emerging epidemic. Clin. Microbiol. Rev. 23 (3), 616–687. doi: 10.1128/CMR.00081-09 20610826 PMC2901661

[B10] David M. Z. Daum R. S. (2017). Treatment of *Staphylococcus aureus* infections. Curr. Top. Microbiol. Immunol. 409, 325–383. doi: 10.1007/82_2017_42 28900682

[B11] Deurenberg R. H. Stobberingh E. E. (2008). The evolution of *Staphylococcus aureus* . Infect. Genet. Evol. 8 (6), 747–763. doi: 10.1016/j.meegid.2008.07.007 18718557

[B12] Elston J. W. Barlow G. D. (2009). Community-associated MRSA in the united kingdom. J. Infect. 59 (3), 149–155. doi: 10.1016/j.jinf.2009.07.001 19619897

[B13] Enright M. C. Day N. P. Davies C. E. Peacock S. J. Spratt B. G. (2000). Multilocus sequence typing for characterization of methicillin-resistant and methicillin-susceptible clones of *Staphylococcus aureus* . J. Clin. Microbiol. 38 (3), 1008–1015. doi: 10.1128/JCM.38.3.1008-1015.2000 10698988 PMC86325

[B14] Fan J. Shu M. Zhang G. Zhou W. Jiang Y. Zhu Y.. (2009). Biogeography and virulence of *Staphylococcus aureus* . PloS One 4 (7), e6216. doi: 10.1371/journal.pone.0006216 19593449 PMC2705676

[B15] Gajdács M. (2019). The continuing threat of methicillin-resistant *Staphylococcus aureus* . Antibio. (Basel). 8 (2), 52. doi: 10.3390/antibiotics8020052 PMC6627156 31052511

[B16] Goering R. V. Shawar R. M. Scangarella N. E. O'Hara F. P. Amrine-Madsen H. West J. M.. (2008). Molecular epidemiology of methicillin-resistant and methicillin-susceptible *Staphylococcus aureus* isolates from global clinical trials. J. Clin. Microbiol. 46 (9), 2842–2847. doi: 10.1128/JCM.00521-08 18614654 PMC2546764

[B17] Gu F. F. Chen Y. Dong D. P. Song Z. Guo X. K. Ni Y. X.. (2016). Molecular epidemiology of *Staphylococcus aureus* among patients with skin and soft tissue infections in two Chinese hospitals. Chin. Med. J. (Engl). 129 (19), 2319–2324. doi: 10.4103/0366-6999.190673 27647191 PMC5040018

[B18] Guo Y. Song G. Sun M. Wang J. Wang Y. (2020). Prevalence and therapies of antibiotic- resistance in *Staphylococcus aureus* . Front. Cell Infect. Microbiol. 10. doi: 10.3389/fcimb.2020.00107 PMC7089872 32257966

[B19] Guo Y. Xu L. Wang B. Rao L. Xu Y. Wang X.. (2023). Dissemination of methicillin-resistant *Staphylococcus aureus* sequence type 764 isolates with mupirocin resistance in China. Microbiol. Spectr. 11 (1), e0379422. doi: 10.1128/spectrum.03794-22 36622214 PMC9927232

[B20] Hashemi H. Mohebbi M. Mehravaran S. Mazloumi M. Jahanbani-Ardakani H. Abtahi S. H. (2017). Hyperimmunoglobulin e syndrome: genetics, immunopathogenesis, clinical findings, and treatment modalities. J. Res. Med. Sci. 22, 53. doi: 10.4103/jrms.JRMS_1050_16 28567072 PMC5426098

[B21] Hassoun A. Linden P. K. Friedman B. (2017). Incidence, prevalence, and management of MRSA bacteremia across patient populations-a review of recent developments in MRSA management and treatment. Crit. Care 21 (1), 211. doi: 10.1186/s13054-017-1801-3 28807042 PMC5557425

[B22] Hesje C. K. Sanfilippo C. M. Haas W. Morris T. W. (2011). Molecular epidemiology of methicillin-resistant and methicillin-susceptible *Staphylococcus aureus* isolated from the eye. Curr. Eye Res. 36 (2), 94–102. doi: 10.3109/02713683.2010.534229 21158584 PMC3021952

[B23] Jin Y Li M. Shang Y. Liu L Shen X. Lv Z.. (2018). Sub-Inhibitory concentrations of mupirocin strongly inhibit alpha-toxin production in high-level mupirocin-resistant MRSA by down-regulating agr, saeRS, and *sarA* . Front. Microbiol. 9. doi: 10.3389/fmicb.2018.00993 PMC5962727 29867891

[B24] Lee G. C. Dallas S. D. Wang Y. Olsen R. J. Lawson K. A. Wilson J.. (2017). Emerging multidrug resistance in community-associated *Staphylococcus aureus* involved in skin and soft tissue infections and nasal colonization. J. Antimicrob. Chemother. 72 (9), 2461–2468. doi: 10.1093/jac/dkx200 28859442 PMC5890715

[B25] Lee A. S. de Lencastre H. Garau J. Kluytmans J. Malhotra-Kumar S. Peschel A.. (2018). Methicillin-resistant *Staphylococcus aureus* . Nat. Rev. Dis. Primers. 4, 18033. doi: 10.1038/nrdp.2018.33 29849094

[B26] Li X. Chen Y. Gao W. Ouyang W. Wei J. Wen Z. (2016). Epidemiology and outcomes of complicated skin and soft tissue infections among inpatients in southern China from 2008 to 2013. PloS One 11 (2), e0149960. doi: 10.1371/journal.pone.0149960 26918456 PMC4769280

[B27] Liu Q. Z. Wu Q. Zhang Y. B. Liu M. N. Hu F. P. Xu X. G.. (2010). Prevalence of clinical meticillin-resistant *Staphylococcus aureus* (MRSA) with high-level mupirocin resistance in shanghai and wenzhou, China. Int. J. Antimicrob. Agents. 35 (2), 114–118. doi: 10.1016/j.ijantimicag.2009.09.018 19939636

[B28] Liu Y. Xu Z. Yang Z. Sun J. Ma L. (2016). Characterization of community-associated *Staphylococcus aureus* from skin and soft-tissue infections: a multicenter study in China. Emerg. Microbes Infect. 5 (12), e127. doi: 10.1038/emi.2016.128 27999423 PMC5180372

[B29] Lowy F. D. (1998). *Staphylococcus aureus* infections. N Engl. J. Med. 339 (8), 520–532. doi: 10.1056/NEJM199808203390806 9709046

[B30] Mediavilla J. R. Chen L. Mathema B. Kreiswirth B. N. (2012). Global epidemiology of community-associated methicillin resistant *Staphylococcus aureus* (CA-MRSA). Curr. Opin. Microbiol. 15 (5), 588–595. doi: 10.1016/j.mib.2012.08.003 23044073

[B31] Miller L. G. Daum R. S. Creech C. B. Young D. Downing M. D. Eells S. J.. (2015). Clindamycin versus trimethoprim-sulfamethoxazole for uncomplicated skin infections. N Engl. J. Med. 372 (12), 1093–1103. doi: 10.1056/NEJMoa1403789 25785967 PMC4547538

[B32] Nakaminami H. Ozawa K. Sasai N. Ikeda M. Nemoto O. Baba N.. (2020). Current status of panton-valentine leukocidin-positive methicillin-resistant *Staphylococcus aureus* isolated from patients with skin and soft tissue infections in Japan. J. Dermatol. 47 (11), 1280–1286. doi: 10.1111/1346-8138.15506 32696497

[B33] Novy P. Kloucek P. Rondevaldova J. Havlik J. Kourimska L. Kokoska L. (2014). Thymoquinone vapor significantly affects the results of *Staphylococcus aureus* sensitivity tests using the standard broth microdilution method. Fitoterapia 94, 102–107. doi: 10.1016/j.fitote.2014.01.024 24508861

[B34] Omuse G. Van Zyl K. N. Hoek K. Abdulgader S. Kariuki S. Whitelaw A.. (2016). Molecular characterization of *Staphylococcus aureus* isolates from various healthcare institutions in Nairobi, Kenya: a cross sectional study. Ann. Clin. Microbiol. Antimicrob. 15 (1), 51. doi: 10.1186/s12941-016-0171-z 27647271 PMC5029008

[B35] Otter J. A. French G. L. (2010). Molecular epidemiology of community-associated meticillin-resistant Staphylococcus aureus in Europe. Lancet Infect. Dis. 10 (4), 227–239. doi: 10.1016/S1473-3099(10)70053-0 20334846

[B36] Rao Q. Shang W. Hu X. Rao X. (2015). *Staphylococcus aureus* ST121: a globally disseminated hypervirulent clone. J. Med. Microbiol. 64 (12), 1462–1473. doi: 10.1099/jmm.0.000185 26445995

[B37] Sahebnasagh R. Saderi H. Owlia P. (2014). The prevalence of resistance to methicillin in *Staphylococcus aureus* strains isolated from patients by PCR method for detection of *mecA* and *nuc* genes. Iran J. Public Health 43 (1), 84–92. 26060684 PMC4454028

[B38] Shallcross L. J. Fragaszy E. Johnson A. M. Hayward A. C. (2013). The role of the panton-valentine leucocidin toxin in staphylococcal disease: a systematic review and meta-analysis. Lancet Infect. Dis. 13 (1), 43–54. doi: 10.1016/S1473-3099(12)70238-4 23103172 PMC3530297

[B39] Turner N. A. Sharma-Kuinkel B. K. Maskarinec S. A. Eichenberger E. M. Shah P. P. Carugati M.. (2019). Methicillin-resistant *Staphylococcus aureus*: an overview of basic and clinical research. Nat. Rev. Microbiol. 17 (4), 203–218. doi: 10.1038/s41579-018-0147-4 30737488 PMC6939889

[B40] Wang X. Shen Y. Huang W. Zhou Y. (2019). Characterisation of community-acquired *Staphylococcus aureus* causing skin and soft tissue infections in a children's hospital in shanghai, China. Epidemiol. Infect. 147, e323. doi: 10.1017/S0950268819002127 31831085 PMC7006014

[B41] Weist K. Cimbal A. K. Lecke C. Kampf G. Rüden H. Vonberg R. P. (2006). Mar;Evaluation of six agglutination tests for *Staphylococcus aureus* identification depending upon local prevalence of meticillin-resistant *S. aureus* (MRSA). J. Med. Microbiol. 55 (Pt 3), 283–290. doi: 10.1099/jmm.0.46225-0 16476792

[B42] Williams D. J. Cooper W. O. Kaltenbach L. A. Dudley J. A. Kirschke D. L. Jones T. F.. (2011). Comparative effectiveness of antibiotic treatment strategies for pediatric skin and soft-tissue infections. Pediatrics 128 (3), e479–e487. doi: 10.1542/peds.2010-3681 21844058 PMC3387880

[B43] Xiao N. Yang J. Duan N. Lu B. Wang. L. (2019). Community-associated *Staphylococcus aureus* PVL+ ST22 predominates in skin and soft tissue infections in Beijing, China. Infect. Drug resist. 12, 2495–2503. doi: 10.2147/IDR.S212358 31616166 PMC6698600

[B44] Yu F. Liu Y. Lv J. Qi X. Lu C. Ding Y.. (2015). Antimicrobial susceptibility, virulence determinant carriage and molecular characteristics of *Staphylococcus aureus* isolates associated with skin and soft tissue infections. Braz. J. Infect. Dis. 19 (6), 614–622. doi: 10.1016/j.bjid.2015.08.006 26408338 PMC9425354

[B45] Zhanel G. G. Adam H. J. Baxter M. Lagace-Wiens P. R. S. Karlowsky J. A. (2021). *In vitro* activity and resistance rates of topical antimicrobials fusidic acid, mupirocin and ozenoxacin against skin and soft tissue infection pathogens obtained across Canada (CANWARD 2007-18). J. Antimicrob. Chemother. 76 (7), 1808–1814. doi: 10.1093/jac/dkab098 33792700

[B46] Zhao R. Wang X. Wang X. Du B. Xu K. Zhang F.. (2022). Molecular characterization and virulence gene profiling of methicillin-resistant staphylococcus aureus associated with bloodstream infections in southern China. Front. Microbiol. 13. doi: 10.3389/fmicb.2022.1008052 PMC9618618 36325019

